# Notes

**Published:** 1899-07-08

**Authors:** 


### NOTES.

The foundation of a new hospital at Boscombe was laid with full Masonic honours last week. The hospital, which is to cost £8,000, is arranged in separate pavilions, with turrets of attractive design.

The prospective hospital for Dundee, which is to be the gift of ex-Provost Moncur, is progressing. A suitable site has been selected, and Mr. E. Cox and Mr. R. Fleming have promised £1,000 each and annual subscriptions of £500 are also secured.

The Bellahouston Dispensary, which is connected with the Glasgow Victoria Infirmary, has been opened recently. The dispensary is built to the memory of the Misses Steven, of Bellahouston, and will form a usual adjunct to the work of the Infirmary.

The foundation-stone of a new cottage hospital was laid at Hanwell on the afternoon of Wednesday, June 21st, by the Countess of Jersey. It was a great event for Hanwell, and much credit is due to those who have spared no pains during the past months to raise the money for the building and to arouse interest in the scheme. At first accommodation for only four beds will be provided, to be extended later as funds permit. The speeches made at the ceremony last week showed that all classes in Hanwell have come forward to help the hospital on; and Lady Jersey, who made a delightful little speech at the close of the proceedings, expressed her willingness to be its patron. The building, which is nearly completed already, will have an attractive exterior. A sum of £50 was presented in purses during the afternoon, and little of the building expenses are left now to be defrayed. But money will be needed to maintain the new hospital in efficient working, and for this the hon. treasurer, Mr. Montagu Sharpe, appealed.

